# Red Brain, Blue Brain: Evaluative Processes Differ in Democrats and Republicans

**DOI:** 10.1371/journal.pone.0052970

**Published:** 2013-02-13

**Authors:** Darren Schreiber, Greg Fonzo, Alan N. Simmons, Christopher T. Dawes, Taru Flagan, James H. Fowler, Martin P. Paulus

**Affiliations:** 1 Department of Politics, University of Exeter, Exeter, United Kingdom; 2 Department of Political Science, Central European University, Budapest, Hungary; 3 Joint Doctoral Program in Clinical Psychology, San Diego State University/University of California San Diego, San Diego, California, United States of America; 4 Department of Psychiatry, University of California San Diego, La Jolla, California, United States of America; 5 Veterans Affairs San Diego Healthcare System, San Diego, California, United States of America; 6 Department of Politics, New York University, New York, New York, United States of America; 7 Laboratory of Biological Dynamics and Theoretical Medicine, University of California San Diego, La Jolla, California, United States of America; 8 Department of Political Science, University of California San Diego, La Jolla, California, United States of America; 9 Division of Medical Genetics, University of California San Diego, La Jolla, California, United States of America; University of Sydney, Australia

## Abstract

Liberals and conservatives exhibit different cognitive styles and converging lines of evidence suggest that biology influences differences in their political attitudes and beliefs. In particular, a recent study of young adults suggests that liberals and conservatives have significantly different brain *structure*, with liberals showing increased gray matter volume in the anterior cingulate cortex, and conservatives showing increased gray matter volume in the in the amygdala. Here, we explore differences in brain *function* in liberals and conservatives by matching publicly-available voter records to 82 subjects who performed a risk-taking task during functional imaging. Although the risk-taking behavior of Democrats (liberals) and Republicans (conservatives) did not differ, their brain activity did. Democrats showed significantly greater activity in the left insula, while Republicans showed significantly greater activity in the right amygdala. In fact, a two parameter model of partisanship based on amygdala and insula activations yields a better fitting model of partisanship than a well-established model based on parental socialization of party identification long thought to be one of the core findings of political science. These results suggest that liberals and conservatives engage different cognitive processes when they think about risk, and they support recent evidence that conservatives show greater sensitivity to threatening stimuli.

## Introduction

A large body of research suggests that liberals and conservatives differ on important psychological characteristics [1]. For example, conservatives demonstrate stronger attitudinal reactions to situations of threat and conflict. In contrast, liberals tend to be seek out novelty and uncertainty [1]. Moreover, Democrats, who are well known to be more politically liberal, are more risk accepting than Republicans, who are more politically conservative [2]. While ideology appears to drive reactions to the environment, environmental cues also influence political attitudes. For instance, external threats prime more conservative attitudes among liberals, moderates, and conservatives [3].

These ideological differences between political partisans have been attributed to logical, psychological, and social constraints [4] and past scholarship has focused primarily on institutional political processes or individual policy preferences, rather than biological differences in evaluative processes. But recent work has revealed physiological correlates of the differential responses to risk and conflict by liberals and conservatives. Consistent with the previously identified attitudinal divergence, conservatives have more intense physical reactions to threatening stimuli than liberals [5]. Conversely, liberals had stronger physiological responses to situations of cognitive conflict than conservatives [6].

Risk taking, the tendency to select an action where there is an uncertain potential for a relatively large beneficial outcome but also the possibility of an adverse outcome [7]–[9] requires balancing conflicting drives to obtain reward and avoid possible losses [10]–[12]. Risk taking is also closely related to and influenced by subjective perception and apprehension of threat [13], [14]. Considering differential physiological responses to threat and conflict by liberals and conservatives, examining neural processes during risk-taking decision-making may be an important avenue for understanding the link between mental processes and political preferences.

The discovery by Kanai and colleagues [15] that four brain regions implicated in risk and uncertainty (the right amygdala, left insula, right entorhinal cortex, and anterior cingulate (ACC)) differed in liberals and conservatives provided further evidence that political ideology might be connected to differences in cognitive processes. In the context of risk-taking decision-making, the amygdala is thought to be important for the processing of affective attributes involved in decision making [16]–[18]. The insular cortex is involved in the representation of internal bodily cues crucial for subjective feeling states and in signaling potential changes in interoceptive state to possible decision-related outcomes [10], [11], [19], [20]. Further, intolerance of uncertainty is related to posterior insula functioning [11]. The ACC is involved in conflict and error monitoring and in action selection [21], [22]. Thus, the regions implicated in risk and conflict, cognitive processes during which liberals and conservatives have been shown to differ in physiological response, are the similar regions shown by Kanai et al. to differ structurally in liberals and conservatives. If patterns of brain activity in these regions during the evaluation of risks could dependably differentiate liberals and conservatives, then we would have further evidence of the link between mental processes and political preferences.

To test a conjecture that ideological differences between partisans reflect distinctive neural processes, we matched publicly available party registration records with the names of participants (35 males, 47 females) who had previously taken part in an experiment designed to examine risk-taking behavior during functional brain imaging. Ideally, we would have also directly inquired about the individuals’ ideological self-identification and attitudes about a set of political issues. However, we were not able to re-contact the participants. While party registration is not a perfect proxy for ideology, a realignment that started in the 1970s has caused the two to become increasingly correlated over the past 40 years [23]. Political polarization at both the mass and elite levels have created a period where ideology and partisanship are substantially overlapping concepts [24]. This trend has been even stronger in California (where the participants in this study resided) than in other states [25].

Individuals completed a simple risk-taking decision-making task [26] during which participants were presented with three numbers in ascending order (20, 40, and 80) for one second each. While pressing a button during the presentation of the number 20 on the screen always resulted in a gain of 20 cents, waiting to select 40 or 80 was associated with a pre-determined possibility of either gaining or losing 40 or 80 cents. Therefore, participants chose between a lower “safe” payoff and a higher risky payoff. The probabilities of losing 40 or 80 cents were calibrated so that there was no expected value advantage to choosing 20, 40 or 80 during the task, i.e. the overall pay-off would have been the same for each pure strategy. Previous studies [26]–[28] using this risk-taking decision-making task found activity in some of the same regions identified by Kanai et al. as differentiating liberals and conservatives.

## Results

**Figure 1:**
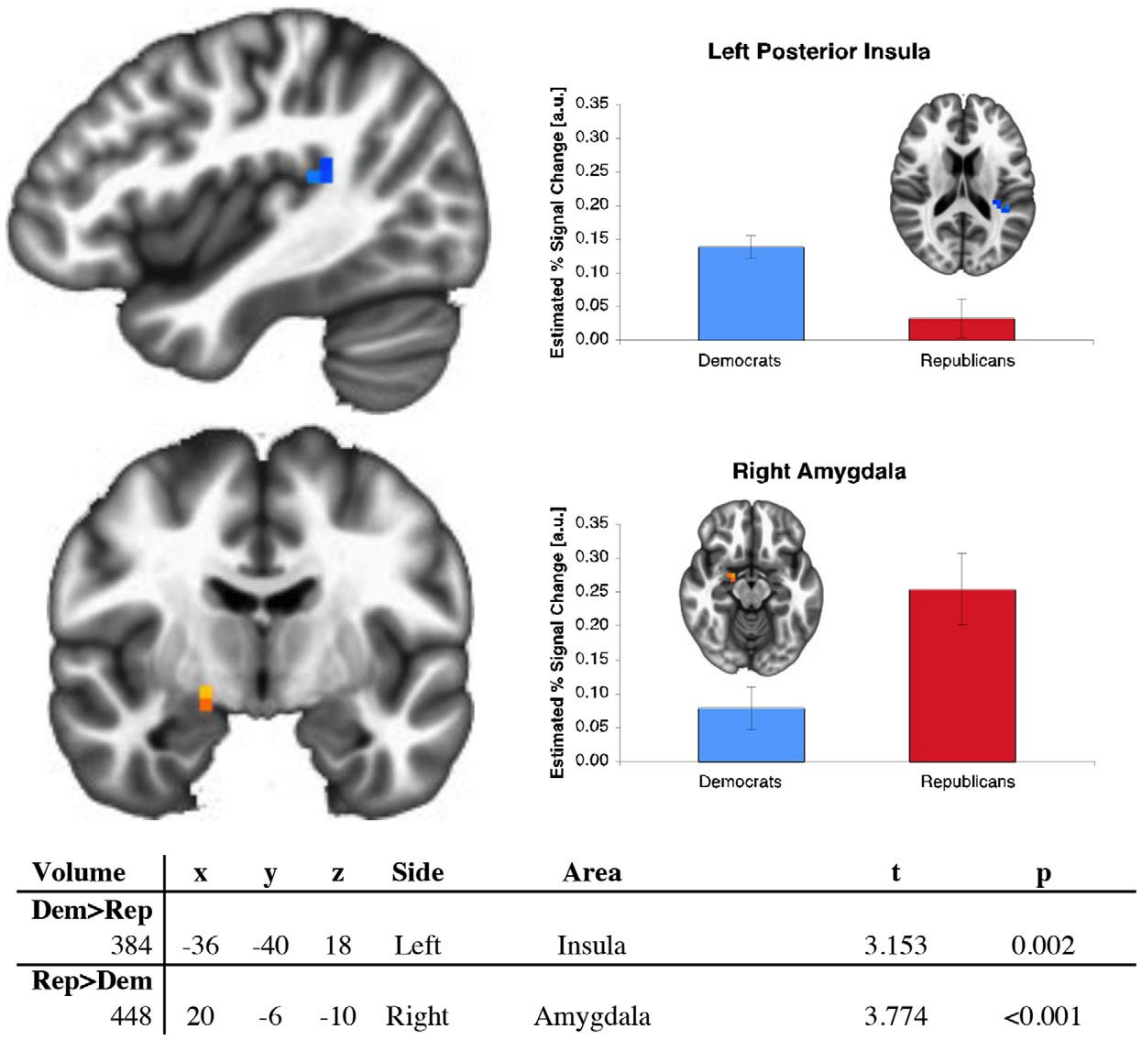
Republicans and Democrats differ in the neural mechanisms activated while performing a risk-taking task. Republicans more strongly activate their right amygdala, associated with orienting attention to external cues. Democrats have higher activity in their left posterior insula, associated with perceptions of internal physiological states. This activation also borders the temporal-parietal junction, and therefore may reflect a difference in internal physiological drive as well as the perception of the internal state and drive of others.

**Figure 2:**
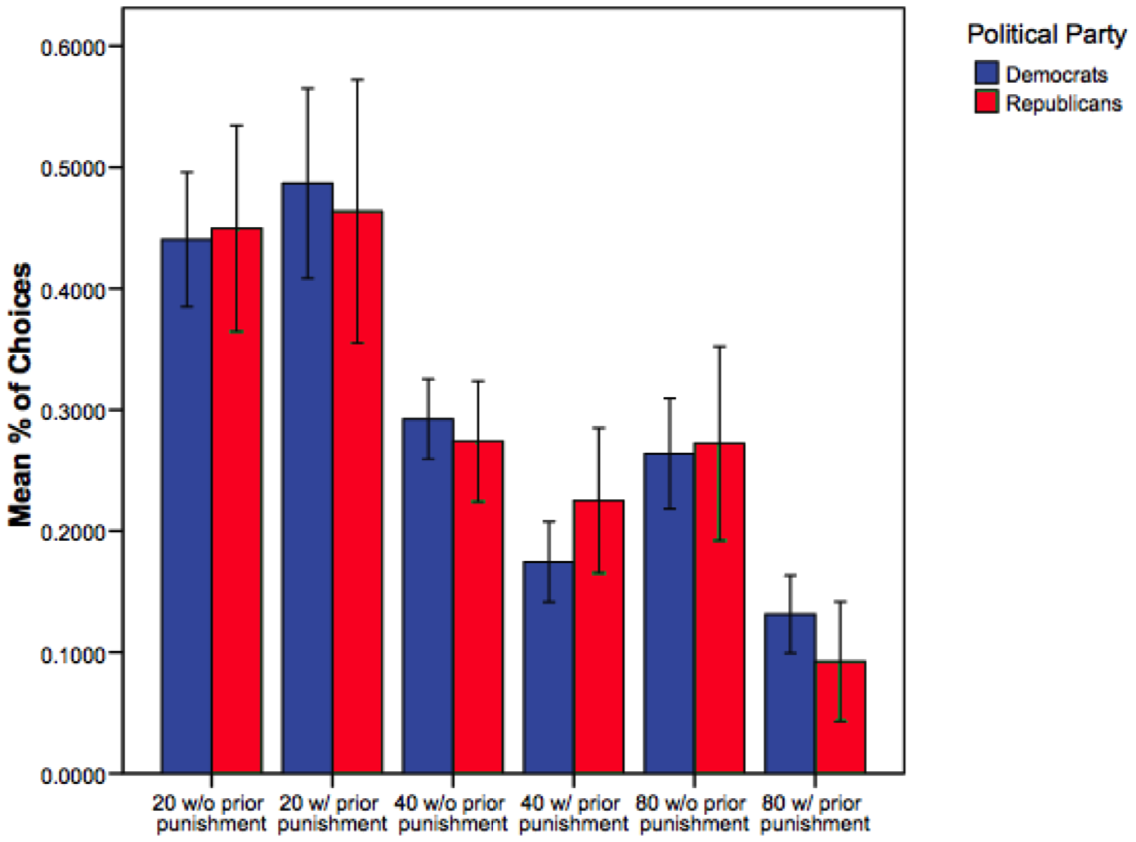
Comparison of behavioral choices in the scanner, by party and decision history. “Punishment” refers to an event in which a subject chose a risky decision and lost. The results show that there were no significant differences in the behavior of Republicans and Democrats.

As an initial test of our conjecture, we examined 5 mm spheres centered on regions in the amygdala, insula, anterior cingulate cortex, and entorhinal cortex that had been previously identified by Kanai et al. [15]. When these specific portions of the regions failed to demonstrate functional differences, we generated larger, anatomically defined masks of the four areas. Consistent with the findings of structural differences by Kanai et al, significantly greater activation was observed in the right amygdala for Republicans and in the left posterior insula (near the temporal-parietal junction) in Democrats when making winning risky versus winning safe decisions (Fig. 1). No significant differences were observed in the entorhinal cortex or anterior cingulate cortex. All attempts to use behavior to distinguish Republicans from Democrats were unsuccessful (Fig. 2), suggesting that different neural mechanisms may underlie apparently similar patterns of behavior [29].

The insula and amygdala often function together in processing situations of risk and uncertainty [30]. The amygdala plays a critical role in orienting of attention to external cues [31] and fear conditioning [32]; however, this structure is also important for other emotional information processing and behavior [33]. Functional neuroimaging studies have shown amygdala activation in reward related processing [34], encoding of emotionally salient information [35], risk-taking [36], processing positively-valenced stimuli [37], and appetitive/aversive olfactory learning [38]. In comparison, neuroimaging studies of insular cortex have observed critical involvement of this neural structure in pain [39], interoceptive [40], emotion-related [41], cognitive [42], and social processing [43]. In particular, the insular cortex is important for representation of internal bodily cues crucial for subjective feeling states and interoceptive awareness [40], [44]. That differences in the processing of risk and uncertainty differentiate liberals and conservatives suggests an alternative way of conceptualizing ideology.

It is important to note the insula region observed in the current study is very posterior and borders the temporal-parietal junction. This region has been conceptualized as vital for “theory of mind” in processing, or the perception of others as thinking entities [45]. In fact a meta-analysis of over 200 fMRI studies on social cognition, the temporal-parietal junction was shown to be related to understanding immediate action intent in others [46]. This suggests that the posterior insula activation found in the current study may reflect internal physiological drive as well as the perception of the internal state and drive of others.

A critical unresolved problem common to studies of the formation of ideology on both individual and institutional levels is the process through which a high dimensional space of distinct values, preferences, or issues is reduced to a low dimensional ideological space [3]. It is even less clear why voters and their representatives in government should organize political attitudes into apparently constrained bundles that are relatively consistent over time [47]. While it has been suggested that biological factors may lead liberals and conservatives to have different sets of politically relevant *values* [48], the evidence presented here suggests that the neural *processes* of evaluation themselves are distinct, perhaps reflecting differentiable values, as well as differing preferences for issues, candidates, and parties.

## Discussion

The strongest finding to come out of the “Michigan school” when the behavioral revolution spread to political science in the 1950s was that parents socialize their children to identify with the same political parties that they do. In fact, the correlation between parent and child is “so familiar and well established” that it is often taken as one of the few “axioms” of political science [49]. Indeed, a simple model of partisanship that includes mother’s and father’s party accurately predicts about 69.5% of self-reported choices between the Democratic and Republican party (see Table S1 in Appendix S1). A classifier model based upon differences in brain *structure* distinguishes liberals from conservatives with 71.6% accuracy [15]. Yet, a simple two-parameter model of partisanship using activations in the amygdala and the insular cortex during the risk task significantly out-performs the longstanding parental model, correctly predicting 82.9% of the observed choices of party (see Table S2 in Appendix S1).

One intriguing remaining puzzle regards the direction of causality. One might infer that the differing brain structures identified by Kanai et al. suggest genetic foundations for the differences in ideology. However, recent work has shown that changes in cognitive function can lead to changes in brain structure [50], [51]. For instance, applicants who worked to learn the map of London in order to pass a knowledge test required of potential cab drivers demonstrated significant growth in their hippocampus, a brain region related to memory formation [52].

Although genetic variation has been shown to contribute to variation in political ideology [48] and strength of partisanship [53], the portion of the variance in political affiliation explained by activity in the amygdala and insula is significantly larger (see Appendix S1), suggesting that acting as a partisan in a partisan environment may alter the brain, above and beyond the effect of the heredity. The interplay of genetic and environmental effects may also be driving the observed correlations between the size of brain regions and political affiliation [15]. Further untangling the roles of party, ideology, genes, and neurocognition will be essential for advancing our understanding of political attitudes and behavior [54]. The ability to accurately predict party identification using only neural activity during a risk-taking task suggests that investigating basic neuropsychological differences between partisans may provide us with more powerful insights than the previously-available traditional tools of psychology, sociology, and political science.

## Materials and Methods

Written informed consent was obtained from all participants and the study was approved by and carried out under the guidelines of the Human Research Protections Program at the University of California, San Diego (UCSD).

Participant groups were composed of 60 Democrats and 22 Republicans who differed with regard to age (F(1,81) = 8.591, p = .004; Democratic mean age  = 22.12 (SD 6.84); Republican mean age  = 28.09 (SD 11.35) (age was therefore entered as a covariate in subsequent analyses to control for any confounding effects), but did not differ in regard to gender (Democrats: 36 females and 24 males; Republicans: 11 females and 11 males; *χ* ^2^ = 2.036, *p* = 0.154.).

The UCSD Institutional Review Board approved study procedures. All participants provided written informed consent and were paid for their participation. We acquired voter registration records from San Diego County in March 2008 that included party of registration and electoral turnout history, and names, addresses, and phone numbers to ensure exact matches to subjects who participated in the functional brain imaging study. Functional imaging data was collected across 1.5T (n = 14) and 3T (n = 68) scanners. There was a difference between Democrats and Republicans on which scanner the data was acquired on (χ^2^ = 78.98, p<.001; Democrats: 5 on 1.5T, 55 on 3T; Republicans: 9 on 1.5T, 13 on 3T). Therefore, the scanner was entered as a covariate to control for confounding effects.

For the Risky-Gains task [26], participants were presented with three numbers in ascending order (20, 40, and 80) in each trial. If the participant pressed a button when the number was shown on the screen, he/she received the number of cents shown on the screen (+20, +40, or +80). The participants were informed that if they pressed the button while the 20 was on the screen, they would always receive 20 cents (safe decision). However, if they waited for the 40 or 80 to appear on the screen, there was a chance the number would appear in red, signaling the loss of 40 or 80 cents, respectively (risky decision). Thus, although the participant may have gained more points per trial by waiting until a 40 or 80 appears on the screen, there was also a risk of losing 40 or 80 points. Participants received feedback (stimulus on the screen and auditory sound) indicating the gain or loss of cents immediately after selecting a response. The probabilities of presenting a negative 40 or 80 are such that a participant's final score would be identical were they to consistently select 20, 40, or 80. Thus, there was no inherent advantage to select the risky response (40 or 80) over the safe response [37]. Each trial lasted 3.5 s irrespective of the participants’ choice. Three trial types were presented in a pseudo-randomized order: non-punished (+20, +40, +80, *n* = 54), punished 40 (−40, *n* = 24), and punished 80 (−80, *n* = 18), along with six null trials that lasted 3.5 s each. Loss of reward only occurred during punished trials, when participants did not respond to the previous numbers on that trial (i.e., did not respond to the 20 on punished 40 trials or did not respond to the 20 or 40 on punished 80 trials).

For 68 participants, during the task a BOLD-fMRI run was collected for each participant using a Signa EXCITE (GE Healthcare, Milwaukee) 3.0T scanner (T2 * weighted echo planar imaging, TR = 2000 ms, TE = 32 ms, FOV = 250×250 mm3, 64×64 matrix, 30 2.6 mm axial slices with a 1.4 mm gap, 290 scans). Functional MRI acquisitions were time-locked to the onset of functional run. During the same experimental session, a high resolution T1-weighted image (SPGR, TI = 450 ms, TR = 8 ms, TE = 4 ms, flip angle = 12°, FOV = 250×250, ∼1 mm3 voxels) was obtained for anatomical reference. For 14 participants, during the task a BOLD-fMRI run was collected for each participant using a 1.5-T Siemens (Erlangen, Germany) scanner (T2*-weighted echo planar imaging, TR = 2,000 ms, TE = 40 ms, 64×64 matrix, 20 4-mm axial slices, 256 repetitions). During the same experimental session, a T1-weighted image (MPRAGE, TR = 11.4 ms, TE = 4.4 ms, flip angle = 10°, FOV = 256×256, 1 mm^3^ voxels) was obtained for anatomical reference.

The data were preprocessed and analyzed with the software AFNI [55]. The echo-planar images were realigned to the temporal center of the longest stable head position and time-corrected for slice acquisition order. To exclude the voxels showing an artifact related to signal drop, a combined threshold/cluster-growing algorithm was applied to the mean of the functional images to compute a region of interest brain mask. This screened out non-brain voxels and voxels falling within the artifact region. A randomized, fast-event related design was used with six resting trials interspersed between the 96 risky-gains trials. The preprocessed time series data for each individual were analyzed using a multiple regression model where five regressors of interest were constructed from the behavioral data obtained from each participant during the task. Specifically, response regressors were defined from the onset of the trial until the individual selected an option and, for punished trials, until the appearance of negative 40 or 80. These five regressors are focused on decisions resulting in a gain of (1) 20 (+20, safe response), (2) 40 (+40, risky response), (3) 80 (+80, risky response), or loss of (4) 40 (−40, risky response), and (5) 80 (−80, risky response). The subsequent time period, which included outcome and intertrial interval, as well as the null trials, served as the baseline condition for this analysis. The regressors of interest were convolved with a modified gamma variate function modeling a prototypical hemodynamic response [56] before inclusion in the regression model. In addition, three regressors were used to account for residual motion (in the roll, pitch, and yaw direction). Regressors for baseline and linear trends were used to eliminate slow signal drifts. The AFNI program 3dDeconvolve was used to calculate the estimated voxel-wise response amplitude. Finally, a participant-specific voxel-based linear contrast was used to identify brain activation associated with selecting a winning risky response (average of +40 or +80, termed win risky) vs a safe response (+20, safe). A Gaussian filter with FWHM 6 mm was applied to the voxel-wise percent signal change data to account for individual variations of the anatomical landmarks. Data of each participant were normalized to Talairach coordinates.

For the Kanai et al. region of interest (ROI) analysis, four 5 mm spherical masks were generated around each of the four locations identified by Kanai et al. [15]: Right Amygdala (16, −4, −34), Left insula (−38, −16, −2), ACC (−3, 24, 25), and Right Entorhinal (22, −21, −26). Average percent signal changes within these ROIs for the win risky versus safe decisions (contrasting regressors 2 and 3 with regressor 1 in the list of regressors given above) were extracted from each subject, and for each ROI these individual extracted values were subjected to a “robust” regression implemented within the statistical package R (www.r-project.org) by modeling effects as a function of age, income, political party, and magnet tesla. The analysis of the specific spheres did not appear statistically significant, so larger ROIs based on the anatomy were used next.

Voxelwise “robust” multiple regression analyses were conducted on individual percent signal change statistics for conditions of interest by modeling effects as a function of age, income, political party, and magnet tesla. A priori regions of interest (ROI) masks (defined by the Talairach Daemon atlas [57]) in the bilateral amygdala, bilateral insula, and anterior cingulate/medial prefrontal cortex (Brodmann Areas 24 and 32), were used to examine between-group effects for the win risky versus safe decisions (contrasting regressors 2 and 3 with regressor 1 in the list of regressors given above). On the basis of these ROIs, a voxel-wise a-priori probability of 0.05 for each model factor, determined via Monte-Carlo simulations, would result in a corrected cluster-wise a posteriori probability of 0.05 with a minimum volume of 192 µl or three connected voxels (in the amygdala) or 320 µl or 5 connected voxels (in all other regions of interest). Using the thresholding and clustering techniques described above, the corrected voxel-wise probabilities are as follows: amygdala *p*<0.0167 and insular cortex *p*<0.01. ROI masks were superimposed on each individual’s voxel-wise percent signal change brain image. Only activations within the areas of interest, which also satisfied the volume and voxel connection criteria, were extracted and used for further analysis. Significance values reported in the cluster table were corrected for age, income, magnet tesla, and gender. Behavioral analyses were carried out with SPSS 12.0 (Chicago, Il).

Several analyses were carried out to determine the degree to which brain activation predicted partisanship. First, receiver-operator characteristic (ROC) curves (See Fig. S1 in Appendix S1) were determined for each functional region of interest as well as for the combination of the two most predictive areas. Second, a step-wise linear discriminant function analysis (F_enter_: p<0.05) was computed with partisanship as the dependent measure and the activation patterns in the areas that differed across democrats and republicans as independent measures. A cross-validation procedure using a leave-one-out classification method (predictions were generated by resampling with one subject removed) was used to determine sensitivity and specificity of the activation patterns to predict partisanship.

## Supporting Information

Appendix S1 (PDF)
