# Supplementary material for: Red Brain, Blue Brain: Evaluative Processes Differ in Democrats and Republicans
Source: PLoS One. 2013 Feb 13;8(2):e52970. doi: 10.1371/journal.pone.0052970 (PMC3572122; doi:10.1371/journal.pone.0052970)
Supplement: Appendix S1 — (PDF) [file pone.0052970.s001.pdf]

SI Appendix for

Red Brain, Blue Brain: Evaluative Processes Differ in Democrats and Republicans

Darren Schreiber[1], Greg Fonzo[2], Alan N. Simmons[3,4], Christopher T. Dawes[1], Taru Flagan[3,5], James H. Fowler[1,6*], Martin P. Paulus[3,4,5]

[1] *Department of Political Science, University of California San Diego, La Jolla, CA 92103 USA*
[2] *Joint Doctoral Program in Clinical Psychology, San Diego State University/University of California-San Diego, San Diego, CA 92120, USA*
[3] *Department of Psychiatry, University of California San Diego, La Jolla, CA 92093, USA*
[4] *Veterans Affairs San Diego Healthcare System, San Diego, CA 92161, USA*
[5] *Laboratory of Biological Dynamics and Theoretical Medicine, University of California San Diego, La Jolla, CA 92093, USA*
[6] *Division of Medical Genetics, University of California San Diego, La Jolla, CA 92093, USA*
*To whom correspondence should be addressed. Email: jhfowler@ucsd.edu

Behavioral Analysis

The average percentage of each participant's choices within each risk condition (20, 40, 80) both following punishment and not following punished trials was subjected to a repeated-measures ANOVA in SPSS 12.0. Risk level (20, 40, and 80) and prior punishment (Yes/No) were entered as repeated measures while political party was entered as a between-subjects factor. There was a significant main effect of risk level ($F(2, 79) = 22.95, p < .001$) such that all participants chose a greater percentage of safe compared to risky trials. There was also a significant main effect of prior punishment ($F(1, 80) = 102.283, p < .001$) such that all participants had a greater percentage of trials following non-punished vs. punished trials. There was also a significant risk level x prior punishment interaction ($F(2, 79) = 12.328, p < .001$) such

that all participants chose a greater percentage of safe trials following punished vs. non-punished trials. The risk level x party, prior punishment x party, and risk level x prior punishment x party interaction terms were not significant (all *p's* > .05), indicating there was no difference between Democrats and Republicans in frequency of risk taking or responses to punishment. These results also held when covarying on age, gender, and income.

Parents and Party Identification

We acquired dataset 4037 from the Inter-Consortium for Political and Social Research (1) about subjects who self-identified either as a Democrat or a Republican in 1997 (v5750), and the partisanship of the parents during the subjects' adolescence, reported by the subjects during early adulthood in 1973 (v584,v590). We conducted a logistic regression and used this to predict the partisanship of the subjects as shown in Table S1. The model correctly predicts the party 69.45% (S.E. 0.55%) of the subjects.

Discriminant Analysis using Amgydala and Insula Activations to Predict Partisanship

In a subsequent step-wise discriminant function analysis to determine whether brain activation patterns related to risk-taking would be useful to predict party affiliation, we found that using a cross-validation method, brain activation in right amygdala and insula correctly predicted the party affiliation of 82.9% of the study participants (for further test details see Table S2). A Receiver Operator Curve revealed that we achieved significantly greater predictive accuracy (AUC = 0.892 +/- 0.035) than chance.

To compare the explanatory power of the amygdala/insula model with estimates from the literature on the biological heredity of political attitudes, ideology, and party affiliation we

generated estimates of the portion of the variance explained using Nagelkerke's pseudo R-squared (0.529). Though imperfectly comparable, the explanatory power of the amygdala/insula model for party affiliation is stronger than the estimated contributions of genetics to party affiliation (0.115), political attitudes (0.421), and ideology (0.46) (2). Thus, even if functional imaging were able to perfectly capture the effect of genetic factors on party identification, the present model still appears stronger than what we would expect to be arising purely from heredity.

Table S1

Independent Variables	*Dependent Variable: Subject party (1=Dem, 0=Rep)* Coefficient	*Standard Error*	*p*
Mother's Partisanship	0.80	0.17	<0.001
Father's Partisanship	0.27	0.17	0.12
Constant	0.00	0.12	0.99
Percent Correctly Predicted	69.45%	0.55%	
Deviance	414.66		
Null Deviance	479.77		
N	347		

Table S2

Crossvalidation Results

Group	*Predicted Democrat*	*Predicted Republican*		
Democrat	85%	15%	*Positive Predictive Value*	0.85
Republican	27.3%	72.7%	*Negative Predictive Value*	0.73
	Sensitivity	*Specificity*	*Correct Prediction*	
	0.757	0.829	81.7%	

Figure S1

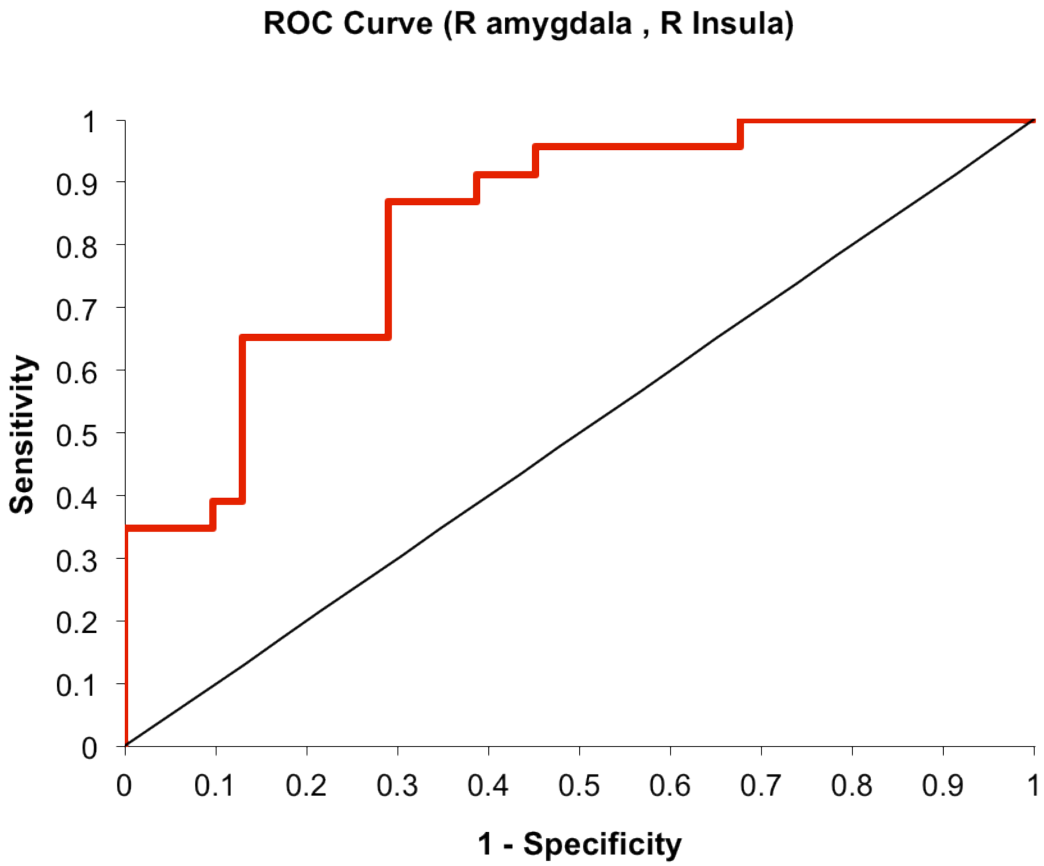

Figure Legends

Figure S1. Receiver operating characteristic (ROC) curve for a model of the left insula and right amygdala predicting political party registration.

References for SI Appendix

1. Jennings MK, Markus GB, Niemi RG, & Stoker L (2005) Youth-Parent Socialization Panel Study, 1965-1997: Four Waves Combined. in *ICPSR04037-v1* (University of Michigan, Center for Political Studies/Survey Research Center).
2. Hatemi PK*, et al*. (2010) Not by twins alone: Using the extended family design to investigate genetic influence on political beliefs. *American Journal of Political Science* 54(3):798-814.
